# Efficiency of a Protective Mode of Mechanical Ventilation in Patients with Severe Traumatic Brain Injury Complicated by Acute Respiratory Distress Syndrome

**DOI:** 10.3390/brainsci15111151

**Published:** 2025-10-27

**Authors:** Marta Rachel, Svitlana Yaroslavska, Konstiantyn Krenov, Maryna Mamonowa, Andriy Dobrorodniy, Oleksandr Oliynyk

**Affiliations:** 1 Department of Allergology and Cystic Fibrosis, Rzeszow University, 35-315 Rzeszów, Poland; mrachel@ur.edu.pl; 2 Department of Anesthesiology and Intensive Care, Bogomolets National Medical University, 01601 Kyiv, Ukraine; kancnmu@nmu.ua (S.Y.); marynamamonova@gmail.com (M.M.); 3 Department of Surgery with a Course in the Basics of Dentistry, Faculty of Postgraduate Education, Vinnytsia National Medical University Named After M. I. Pirogov, Pilotna St. 1, 29000 Khmelnytski, Ukraine; xol.incoming@gmail.com; 4 Department of Anesthesiology and Intensive Care, Ternopil National Medical University, 46000 Ternopil, Ukraine; medicaldepartment@tdmu.edu.ua

**Keywords:** severe traumatic brain injury, acute respiratory distress syndrome, protective lung ventilation

## Abstract

Background/Objectives: Treatment of severe traumatic brain injury (TBI) remains a major challenge in neurocritical care. The functional state of the brain largely depends on the applied ventilation strategy. Many patients develop acute respiratory distress syndrome (ARDS), for which lung-protective ventilation is recommended. However, its effect on outcomes in severe TBI remains unclear. This study aimed to assess whether a lung-protective ventilation strategy improves short-term outcomes in patients with severe TBI complicated by ARDS. Methods: This multicenter retrospective study included patients with severe TBI and ARDS treated in three Ukrainian tertiary hospitals. Lung-protective ventilation was defined as the use of a low tidal volume and moderate positive end-expiratory pressure (PEEP). The primary endpoint was 28-day mortality; secondary endpoints included the Glasgow Coma Scale (GCS) score and intracranial pressure (ICP) on day 28. Univariate and multivariate logistic regression analyses identified factors associated with mortality. Results: Mortality did not depend on arterial PaO_2_ (*p* = 0.173) but correlated with lower GCS (*p* < 0.001), reduced PaO_2_/FiO_2_ ratio (*p* < 0.001), higher tidal volume (*p* < 0.001), and lower PEEP (*p* < 0.001). Lung-protective ventilation reduced mortality from 78.6% to 31.4%. Conclusions: Lung-protective ventilation is safe and effective in severe TBI with ARDS, significantly improving short-term survival without compromising cerebral outcomes.

## 1. Introduction

Traumatic brain injury (TBI) is a leading cause of death and disability worldwide [1,2]. Every year, 50 to 60 million new cases of TBI are reported worldwide, creating a substantial public health burden [3]. Severe TBI has a mortality rate of approximately 40% and can cause significant physical, psychosocial, and social impairment in 60% of patients [4,5]. Patients with severe TBI experience respiratory failure due to loss of protective airway reflexes and decreased respiratory activity. They are at risk of pulmonary complications, primarily ventilator-associated pneumonia and acute respiratory distress syndrome (ARDS) [6]. Patients with severe TBI require mechanical ventilation (MV) [4]. MV is of great importance for their treatment since, in addition to ensuring oxygen delivery to tissues, it also helps to modulate the reactivity of cerebral vessels [5,7]. The benefits of MV come at a price, as its use significantly increases the risk of developing pneumonia and ARDS [8]. Currently, intensive care for patients with severe TBI is aimed at maintaining physiological parameters that can minimize secondary brain damage. This damage is triggered by ischemia resulting from decreased cerebral blood flow [9]. Oxygen supplementation is one of the cornerstones of TBI treatment. The relationship between hyperoxia and outcomes still needs to be explored [10]. It remains unclear what target oxygenation should be aimed at in these patients [11]. Principles well established in normotensive care conflict with lung protective strategies that aim to reduce lung injury caused by forced MV [6].

There are no clear recommendations regarding selecting the optimal ventilation mode in patients with severe TBI [12]. The latest update to the International Guidelines for treating TBI does not explicitly address MV, and the only factor mentioned is strict PaCO_2_ control to avoid hypercapnia. Modifying existing respiratory therapy strategies in patients with severe TBI is crucial because it affects the outcome of the disease. A European Society of Intensive Care Medicine study showed that in patients with TBI, MV methods and parameters vary greatly between centers and depend significantly on local policy and clinical practice [6,13]. Diagnostic and therapeutic studies are needed to improve treatment outcomes [14]. Despite the high incidence of pulmonary complications, there is still a lack of evidence-based recommendations for optimal ventilation management in patients with severe traumatic brain injury complicated by acute respiratory distress syndrome (ARDS). Existing guidelines focus primarily on maintaining normocapnia and cerebral perfusion, but they do not address the interaction between lung-protective ventilation and intracranial dynamics. Protective ventilation in patients with severe TBI represents a physiological challenge, as low tidal volume and higher PEEP can influence cerebral perfusion pressure and intracranial dynamics. We hypothesized that a balanced lung-protective strategy—combining low tidal volume and moderate PEEP—could improve survival in patients with TBI complicated by ARDS by optimizing oxygenation and maintaining stable intracranial pressure.

Thus, the present study was designed to determine whether a lung-protective ventilation approach—combining low tidal volume and moderate PEEP—could improve survival in patients with severe TBI complicated by ARDS while maintaining stable intracranial parameters. Therefore, the present study aimed to evaluate the impact of different mechanical ventilation strategies on respiratory and neurological outcomes in patients with severe TBI complicated by ARDS, with particular attention to PaCO_2_, PEEP levels, and intracranial pressure.

## 2. Methods

### 2.1. Study Design

This was a retrospective multicenter observational study based on medical record review performed between September 2022 and April 2024. The study included adult patients with severe traumatic brain injury (TBI) complicated by acute respiratory distress syndrome (ARDS) who were treated in intensive care units (ICUs) of three Ukrainian tertiary hospitals (Kyiv City Hospital No. 4, Ternopil Emergency Medicine Hospital, and Khmelnytsky Regional Hospital).

Inclusion criteria were: (1) age ≥ 18 years; (2) severe traumatic brain injury (GCS ≤ 8); (3) diagnosis of ARDS according to the Berlin criteria [15]; and (4) availability of complete ventilation and outcome data.

Exclusion criteria were: (1) chronic pulmonary disease; (2) pregnancy; and (3) missing essential data on ventilation parameters or outcomes. A total of 307 patients were initially screened, of whom were excluded due to chronic obstructive pulmonary disease. The final sample consisted of 301 cases.

Patients were excluded to avoid confounding effects on gas exchange and intracranial pressure measurements. Before the start of the study, the partial pressure of oxygen (PaO_2_) and carbon dioxide (PaCO_2_) in the blood was determined using ABL800 FLEX devices from Radiometer, Denmark, and intracranial pressure using Integra Neurosciences MRM-1 devices, Bimedis, Japan. Daily monitoring of arterial PaCO_2_ levels and intracranial pressure was conducted for all patients. ICP was continuously monitored using intraparenchymal probes (Codman ICP Express) and averaged over 24 h periods. These parameters were recorded in clinical documentation and used to guide dynamic assessment and treatment adjustment.

The primary endpoint of the study: 28-day mortality. Secondary endpoints of the study: The condition on day 28, according to the Glasgow Coma Scale; ICP on day 28. Two modes of ventilation were compared: protective and non-protective.

### 2.2. Data Collection

All consecutive eligible cases were included. Data were collected retrospectively from electronic and physical medical records using a standardized form developed before data extraction. In Ukraine, all inpatient medical records are maintained in an electronic format and transmitted to a centralized national server located in Kyiv. All study centers used the EMCiMED Medical Electronic System, which ensures unified data structure and terminology across institutions, minimizing variability and transcription errors. All entries were independently verified by two investigators.

Data collection was performed by three trained researchers who had no involvement in patient treatment. No double-review process was applied, but all data collectors received prior training and used identical extraction procedures. Data were retrieved chronologically according to predefined variables (demographic, clinical, and ventilatory parameters). Investigators were blinded to patient outcomes at the time of data extraction to minimize ascertainment bias.

To further reduce bias, the following strategies were implemented:

− Standardized protocol with clear variable definitions shared by all centers (Table 1).
− Independent data extraction in each hospital.
− Chronological order of extraction without knowledge of outcomes.
− Random cross-checking of 10% of records by an independent researcher.
− Inclusion of only numerically documented data, excluding subjective estimates.

This table summarizes the unified treatment standards used in all centers, including ventilation, ICP management, and sedation. Minor variations between centers were limited to initial FiO_2_ and weaning strategies.

### 2.3. Mechanical Ventilation and Classification of Modes

Mechanical ventilation was performed using Hamilton C6 ventilators (Hamilton Medical AG, Bonaduz, Switzerland) in volume-controlled mode. Ventilatory parameters (tidal volume, PEEP, FiO_2_, plateau pressure, and peak inspiratory pressure) were automatically recorded in the electronic medical record each time the ventilator settings were adjusted and verified daily by attending physicians.

Protective ventilation was defined as tidal volume (Vt) < 8 mL/kg ideal body weight (IBW) and PEEP > 5 cm H_2_O. In this case Pplat did not exceed 30 cm H_2_O. Non-protective ventilation included all other modes not meeting these criteria, specifically those with tidal volume ≥ 8 mL/kg of ideal body weight and/or PEEP ≤ 5 cm H_2_O. For the analysis, only explicitly documented numerical parameters from electronic records were used; no estimated or subjective data were included. All parameter changes were automatically time-stamped and archived in the EMCiMED system, ensuring traceability and accuracy.

### 2.4. Standardized Treatment Protocol

A standardized protocol was used in all participating ICUs. The main treatment approaches are summarized in Table 1, including mechanical ventilation settings, ICP management, fluid therapy, hemodynamic support, sedation and analgesia, antibiotic prophylaxis, and ARDS management. Minor inter-center variations were limited to initial FiO_2_ settings (0.4–0.5) and weaning procedures depending on the attending physician’s discretion.

### 2.5. Sample Size Calculation

As this was a retrospective multicenter study, no formal sample size calculation was performed. All consecutive eligible patients were included, which maximized representativeness and reduced selection bias. A post hoc power analysis demonstrated sufficient statistical power (>80%) for the main outcome (28-day mortality).

### 2.6. Ethical Considerations and Patient Confidentiality

Patient confidentiality was maintained in accordance with institutional and national regulations. All data were anonymized before analysis, and identifiers (name, date of birth, address, medical record number) were removed. Each center provided de-identified datasets containing coded patient numbers. Access to the database was limited to authorized investigators. All files were stored on password-protected institutional computers. The study complied with GDPR principles, the Declaration of Helsinki, and Ukrainian data protection laws.

Because this was a retrospective, non-interventional study, informed consent was not required under Ukrainian law. No refusals were recorded. The study was approved by the local ethics committee (Protocol No. 239, Ternopil National Medical University, 18 August 2022).

### 2.7. Follow-Up and Outcomes

All patients were followed for 28 days after ICU admission or until discharge or death, whichever occurred first. No patients were lost to follow-up. Data on 28-day survival, Glasgow Coma Scale (GCS), and intracranial pressure (ICP) were available for all cases. Minor missing intermediate values (<3%) were replaced using mean substitution within each patient’s record.

### 2.8. Handling of Missing Data and Outliers

Before statistical analysis, all variables were screened for completeness and outliers. Missing data were minimal (<5%) and were handled as follows: continuous variables were imputed using column mean substitution; categorical variables with missing values were excluded from multivariate models but retained in descriptive statistics. Missing data (range 0–4.7%) were mainly related to arterial blood gases. Outliers were identified using interquartile range (IQR) and z-score methods. Implausible values were verified against the original records and excluded if not confirmed. Sensitivity analysis confirmed that the exclusion of outliers did not materially affect model results.

Across all participating centers, treatment protocols were standardized according to national neurocritical care recommendations. Although minor differences in individual management (such as sedation depth, osmotherapy adjustments, or ICP-targeted therapy) could occur, these aspects followed the same clinical algorithms and monitoring criteria. This standardization minimized inter-center variability and reduced potential confounding effects on outcomes.

### 2.9. STROBE Statement

**Figure 1:**
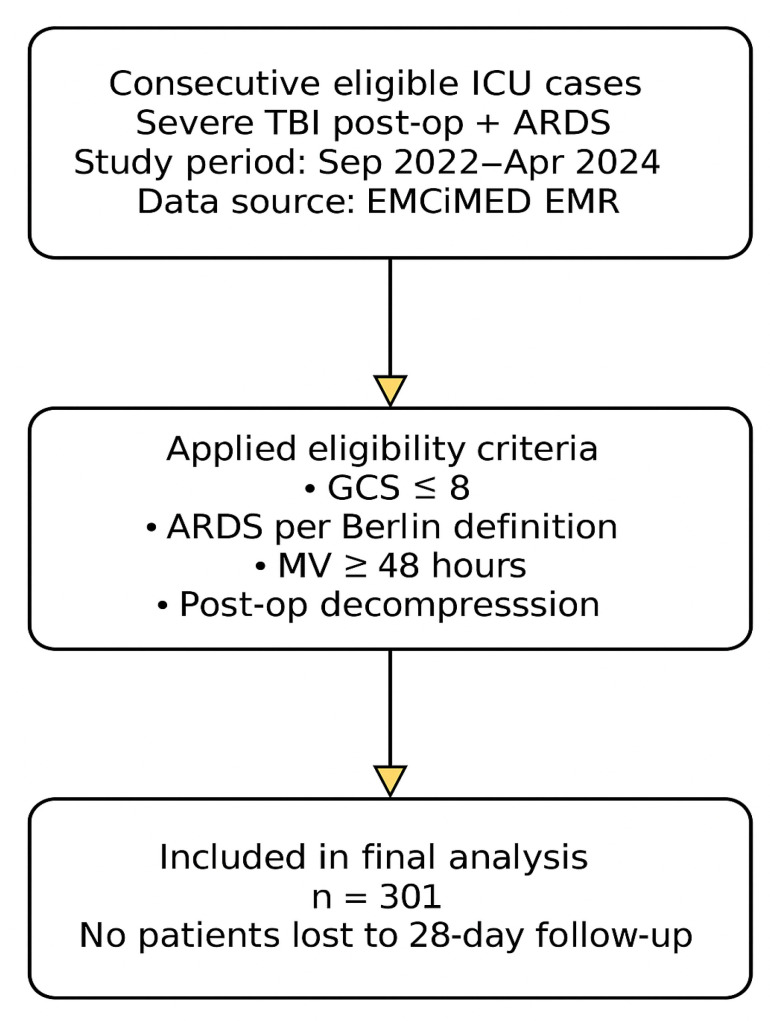
Flowchart of patient selection and inclusion in the study according to STROBE guidelines.

This study was designed and reported in accordance with the STROBE (Strengthening the Reporting of Observational Studies in Epidemiology) guidelines for observational studies (Figure 1) [16].

Figure 1 presents the patient inclusion process according to STROBE guidelines. A total of 301 consecutive ICU cases meeting the eligibility criteria were analyzed.

### 2.10. Statistical Analysis

MedCalc® Statistical Software version 20.009 (MedCalc Software Ltd., Ostend, Belgium; https://www.medcalc.org; 2023, accessed on 15 July 2023) was used in the analysis. If the distribution was non-normal by the Shapiro–Wilk test, the median (Me) and interquartile range (QI–Q3) were calculated to represent quantitative data, and the Mann–Whitney test was used for comparison. Multicollinearity was assessed using variance inflation factor (VIF), and all included predictors had VIF < 2.0. Occurrence (%) was calculated for qualitative variables. Fisher’s exact test was used to compare qualitative characteristics. To evaluate the effect of factors on the studied phenomenon, logistic regression analyses were used, and odds ratios (OR) with a 95% confidence interval (CI) were calculated. The area under the curve (AUC) of the receiver operating characteristic (ROC) and its 95% CI were calculated to assess the model’s predictive accuracy. Youden’s Index was used to calculate the cut-off value of the model. The corresponding sensitivity (with 95% CI), specificity (with 95% CI), and possibly the positive and negative predictive values (+PV and −PV, respectively) were calculated. As this was a retrospective multicenter study, no formal a priori sample size calculation was performed. However, a post hoc power analysis indicated that the sample of 301 patients provided >80% power to detect a 20% difference in 28-day mortality between ventilation strategies at a significance level of 0.05. All statistical tests were two-tailed, and a *p*-value < 0.05 was considered statistically significant.

Logistic regression coefficients were calculated with the assistance of ChatGPT (OpenAI, GPT-5, 2025 version), an artificial intelligence language model, which was used to verify formula implementation and computational logic.

The study was carried out as part of the research work “Improving existing and newly developed methods of prevention, diagnosis, and treatment of the most common and socially significant diseases,” state registration number 0119U002307 of Ternopil National Medical University, Ukraine. To plan the work, consent was obtained from the Biological Ethics Committee of the Ternopil National Medical University, No. 239 dated 18 August 2022.

## 3. Results

Some demographic indicators, respiratory index, and indicators of the functional state of the central nervous system before the start of the analyzed ventilation period were shown in Table 2. We confirmed that no patients were lost to follow-up during the 28-day observation period.

The table presents descriptive statistics of respiratory and neurological parameters in the study cohort, illustrating the variability of gas exchange and ventilation settings among patients with severe TBI complicated by ARDS.

The study included 301 patients: 121 (40.2%) received protective ventilation (Vt < 8 mL/kg IBW, PEEP > 5 cm H_2_O), and 180 (59.8%) were managed with non-protective settings. Among the analyzed 301 patients, 50 developed sepsis during ICU stay; these cases were included in the overall cohort and analyzed within the same regression models. The mortality rate in this subgroup was 72.5%, consistent with the overall trend of higher mortality among patients with multiple organ dysfunction.

As evidenced by PaCO_2_ indicators, patients were ventilated in normal ventilation mode. The ventilation modes used did not significantly affect the level of ICP and neurological status of the patient. We clarified that ventilation did not affect ICP or neurological status was an observational finding, not a causal conclusion. 

Table 3 summarizes key respiratory and neurological parameters recorded during mechanical ventilation in patients with severe TBI and ARDS, providing an overview of gas exchange efficiency and ventilatory settings within the study population.

Between-group differences in continuous variables (ICP, PaCO_2_, PEEP) were tested using the Mann–Whitney U-test. No significant differences in ICP or Glasgow Coma Scale scores were found between the protective and non-protective ventilation groups (median ICP 18 [IQR 15–21] vs. 17 [IQR 14–22] mm Hg; median GCS 6 [IQR 4–8] vs. 6 [IQR 4–8]; *p* = 0.41 and *p* = 0.57, respectively (Table S1). Between-group differences in intracranial pressure and neurological status were formally assessed using the Mann–Whitney U-test. Median intracranial pressure and Glasgow Coma Scale values did not differ significantly between the protective and non-protective ventilation groups (*p* > 0.05). These findings indicate that the type of ventilation strategy did not influence cerebral dynamics or short-term neurological recovery. Higher PaCO_2_ levels were independently associated with increased mortality (β > 0, *p* < 0.05). No variable had more than 5% missing values.

Table 4 shows the distribution of mortality according to ARDS severity and ventilation mode, illustrating the relationship between respiratory status and clinical outcomes in the study population. Protective ventilation was associated with a significantly lower 28-day mortality compared to non-protective ventilation (31.4% vs. 78.6%; OR = 0.13, 95% CI 0.08–0.21, *p* < 0.001.

A univariate logistic regression model was performed to analyze the mortality risk. Mortality depended on Glasgow1, PaO_2_/FiO_2_, FiO_2_, Vt, PEEP, ICP1. In all cases, *p* < 0.001 (Table 5). We specified that the regression model showed good discrimination (AUC) and calibration, and that missing values were <5% and imputed without impact on results.

**Figure 2:**
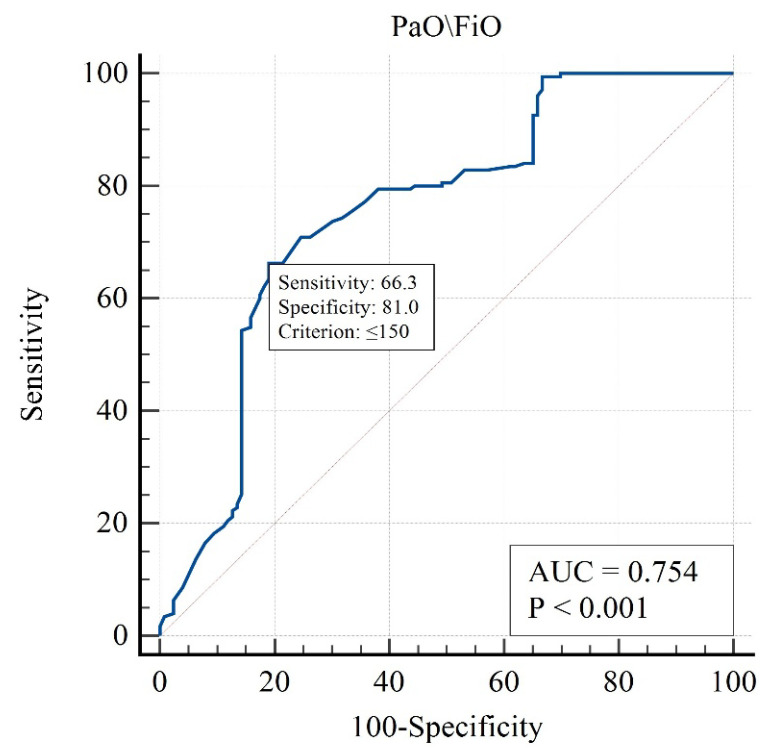
ROC curve for PaO_2_/FiO_2_ ratio in predicting ICU mortality.

**Figure 3:**
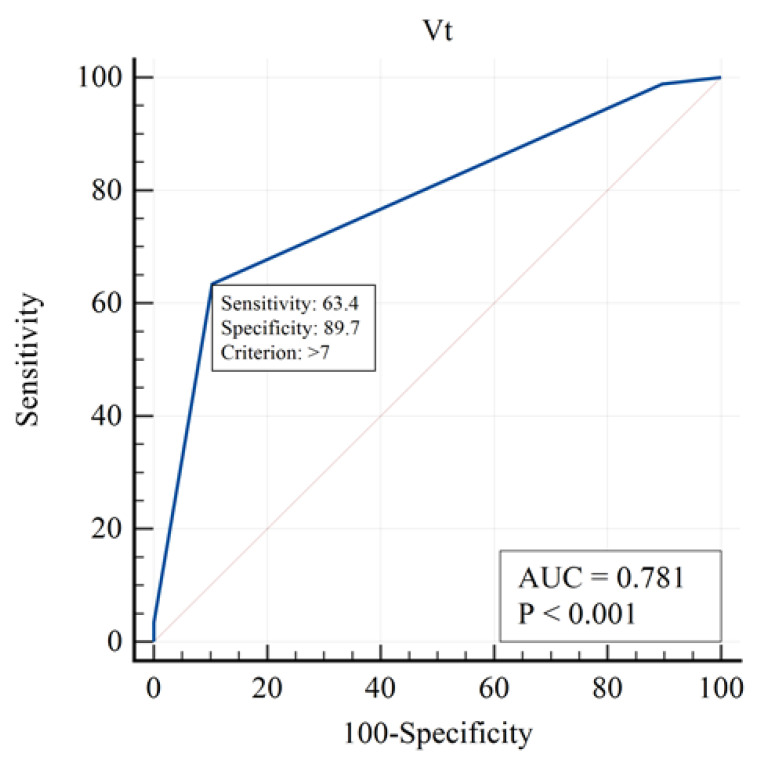
ROC curve for tidal volume (Vt) in predicting ICU mortality.

**Figure 4:**
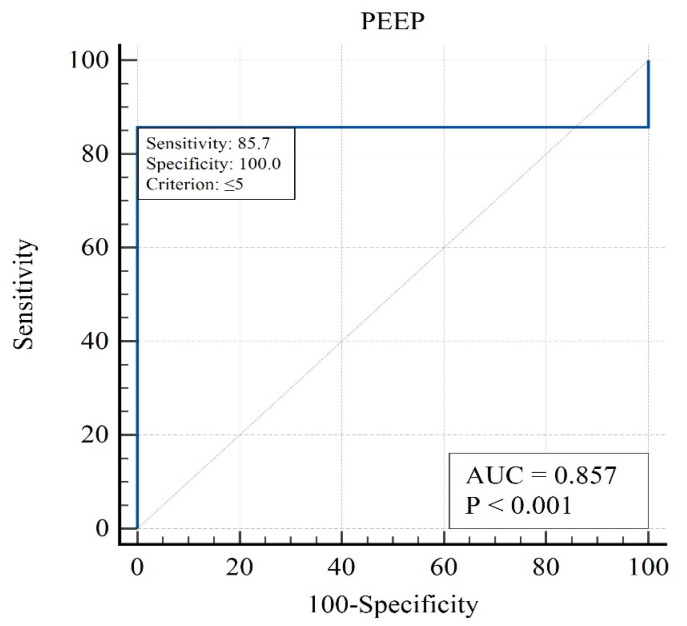
ROC curve for positive end-expiratory pressure (PEEP) in predicting ICU mortality.

This table presents the contribution of each variable to mortality prediction. PaO_2_/FiO_2_, PEEP, and GCS were among the strongest independent predictors. Mortality was not significantly associated with PaO_2_ (OR = 0.99, 95% CI 0.98–1.00, *p* = 0.173; Table 5), indicating that variations in arterial oxygen levels did not independently influence survival outcomes. The analysis for sodium concentration, osmolarity, peak inspiratory pressure, and plateau pressure could not be performed due to insufficient variability in these parameters, which limited their discriminative capacity in the logistic regression model. The strongest influence in the one-factor approximation was for the following factors (Figure 2, Figure 3 and Figure 4):

Figure 2 illustrates the patient selection flow and study grouping. A higher PaO_2_/FiO_2_ ratio is associated with improved survival, confirming its predictive value for mortality in patients with TBI and ARDS.

The curve demonstrates that higher tidal volumes increase mortality risk, supporting the use of lung-protective ventilation strategies.

The analysis highlights that moderate PEEP levels are associated with the lowest mortality, identifying PEEP as a modifiable therapeutic target.

**Figure 5:**
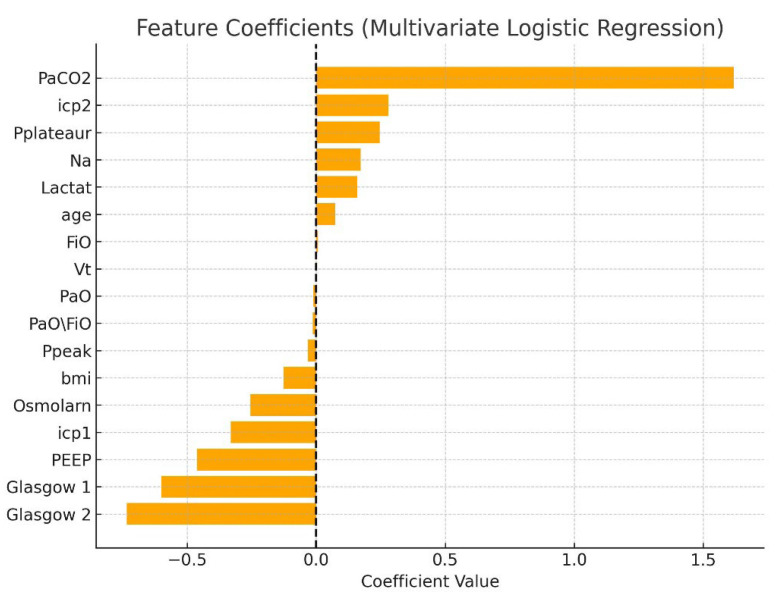
Feature importance plot of machine learning model predictors.

**Figure 6:**
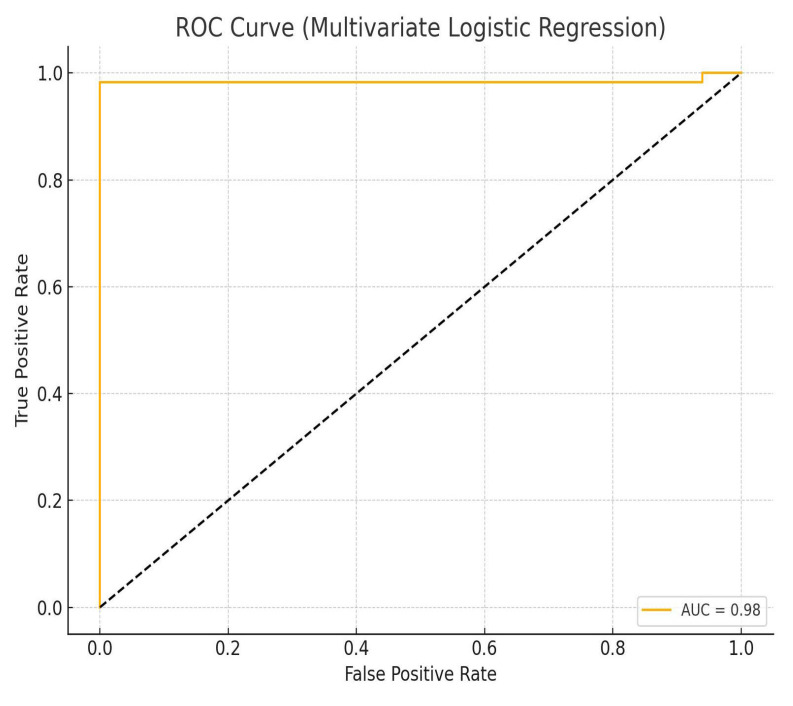
ROC curve for the final predictive model including combined variables.

A multivariate logistic regression model was constructed to assess factors associated with mortality in patients with TBI complicated by ARDS. The logistic regression model demonstrated acceptable discrimination (AUC = 0.79). The analysis included the following variables: age, BMI, PaO_2_/FiO_2_ ratio, tidal volume (Vt), positive end-expiratory pressure (PEEP), intracranial pressure at two stages (icp1, icp2), Glasgow Coma Scale scores (G1, G2), arterial PaCO_2_, lactate, serum sodium (Na), osmolarity, peak inspiratory pressure (Ppeak), and plateau pressure (Pplateau). All variables were standardized, and missing data were imputed with column means. The target variable was in-hospital mortality (Death = 1, Live = 0). The logistic regression model demonstrated a high discriminatory capacity with an area under the ROC curve (AUC) of 0.86. Key predictors of mortality included elevated PaCO_2_ (β = +2.59), lower PEEP (β = −1.43), lower initial GCS (β = −0.61), elevated tidal volume (Vt), and higher ICP at the second stage (icp2). Elevated serum lactate and sodium levels were also associated with increased risk. The model’s coefficients are presented in Table 6, and corresponding graphical representations are shown in Figure 5 and Figure 6.

This plot visualizes the relative influence of clinical and ventilatory variables on mortality risk, with PaCO_2_ and ICP emerging as the most significant factors.

The model demonstrates high discriminatory capacity (AUC = 0.86), confirming good overall predictive performance for mortality outcomes.

Protective ventilation with moderate PEEP and controlled PaCO_2_ was associated with more favorable short-term outcomes and stable intracranial parameters in patients with severe TBI and ARDS. The combined analysis of PaCO_2_, PEEP, ICP, and GCS demonstrated predictive value for survival. These results support the feasibility and safety of lung-protective ventilation in neurocritical care patients with concurrent respiratory failure.

## 4. Discussion

In our study, we did not assess patients using the Lung Injury Prediction Score (LIPS), including the LIPS-2011 version, because this score is not intended for evaluating patients with established ARDS. Instead, it is designed to predict the risk of developing ARDS in hospitalized patients with predisposing conditions. LIPS is applied prior to the onset of ARDS to identify high-risk individuals who may benefit from preventive strategies.

The present findings support the safety of lung-protective ventilation in patients with severe TBI complicated by ARDS, indicating that moderate levels of PEEP and lower tidal volumes do not adversely affect intracranial pressure or neurological status. This suggests that clinicians can apply protective ventilation strategies without compromising cerebral dynamics, thereby reducing ventilator-associated lung injury.

The results of multivariate analysis support existing clinical knowledge regarding the prognostic importance of ventilatory parameters and neurological status in patients with TBI and ARDS. Hypercapnia, metabolic acidosis (elevated lactate), and increased ICP are strong indicators of poor outcome. Conversely, adequate PEEP levels and preserved Glasgow Coma Scale (GCS) scores are predictive of favorable treatment outcomes. Importantly, no critical multicollinearity was found between predictors, validating the integrity of the model.

Elevated PaCO_2_ and low PEEP levels were identified as modifiable parameters associated with poor outcomes. These findings emphasize the importance of maintaining normocapnia and applying moderate PEEP levels to optimize gas exchange without compromising intracranial pressure in patients with severe TBI and ARDS. The observed association between elevated PaCO_2_ and unfavorable outcomes underlines the importance of maintaining strict normocapnia in neurocritical care. Even moderate hypercapnia can lead to cerebral vasodilation, increased intracranial pressure, and secondary ischemia, which are detrimental in patients with TBI. Conversely, excessive hypocapnia should also be avoided, as it may cause cerebral vasoconstriction and reduced cerebral perfusion. Therefore, careful control of PaCO_2_ remains a clinically modifiable target with direct relevance to patient management.

Similarly, the results emphasize the role of moderate positive end-expiratory pressure (PEEP) as a potentially protective ventilation parameter. While high PEEP may theoretically increase ICP through elevated intrathoracic pressure, our findings support that moderate PEEP levels (6–10 cm H_2_O) can improve oxygenation without worsening intracranial dynamics. These insights highlight the feasibility of balancing lung-protective ventilation with neuroprotection in patients with severe TBI complicated by ARDS.

According to current international guidelines, strict control of PaCO_2_ is recommended to prevent secondary brain injury, while both hypercapnia and hypocapnia may negatively affect cerebral hemodynamics and outcomes. In our study, PaCO_2_ and PEEP emerged as key modifiable parameters influencing survival, highlighting the importance of maintaining normocapnia and moderate PEEP levels in patients with severe TBI complicated by ARDS.Therefore, PaCO2 must be maintained at the average level [17,18,19]. Hypocapnia increases cerebral ischemia. Thus, using ventilation modes that include hypocapnia is currently not recommended [20].

MV may harm the brain due to complex physiological interactions between the intrathoracic, central venous, and intracranial regions [4,14]. Traditionally, low PEEP and high Vt (10 mL/kg or higher) have been used to control PaCO_2_ in patients with TBI [21]. However, such regimens may cause respiratory complications, primarily ARDS [11]. The risk of ARDS in patients with TBI is also increased due to post-traumatic autoimmune and immune damage to the lungs, neurogenic pulmonary edema, and impaired pulmonary defense mechanisms due to impaired consciousness [22]. Thus, in the case of TBI complicated by ARDS, the provision of respiratory support is significantly complicated. Overall, our findings indicate that the optimal ventilation strategy for patients with severe TBI complicated by ARDS has yet to be fully developed [6,11,14]. Recommendations regarding the ventilation of patients with severe TBI complicated by ARDS are controversial. On the one hand, a systematic review and meta-analysis by Karim Asehnoune, 2023 [4] shows that there were no differences in mortality in patients with severe TBI depending on the use of low or high Vt [OR 0.88 (95% CI 0.74 to 1.05), *p* = 0.16], from low, moderate or high PEEP [OR 0.8 (95% CI 0.59 to 1.07); *p* = 0.13] or the use of a protective ventilation mode [OR 1.03 (95% CI 0.93 to 1, 15), *p* = 0.6]. In addition, the values of Vt [OR 0.74 (95% CI 0.45 to 1.21); *p* = 0.23] and PEEP [OR 0.98 (95% CI 0.76 to 1.26); *p* = 0.9], as well as the fact of using protective ventilation [OR 1.22 (95% CI 0.94 to 1.58), *p* = 0.13] did not affect the incidence of ARDS.

Recent European Society of Intensive Care Medicine (ESICM) recommendations for MV in patients with TBI without ARDS suggest that PEEP should be set according to the same principles considered in the general ICU population [23]. According to other authors, using low Vt and high PEEP, a protective ventilation strategy, does not improve survival in patients with severe TBI [24,25]. Elevated levels of PEEP can reduce systemic venous return, mean arterial pressure, and cerebral perfusion pressure, which can have detrimental consequences on cerebral blood flow, mainly in cases of impaired cerebral autoregulation [6]. The traditional use of low levels of PEEP (≤5 mmHg) in patients with TBI is explained not only by the positive effect on hemodynamics but also by the compliance of the respiratory system [21]. Respiratory compliance influences ICP and cerebral circulation [26]. In patients with low compliance, an increase in PEEP to 10–12 cm wg does not cause an increase in ICP; in contrast, patients with higher PEEP experience pulmonary over-distension, decreased cerebral venous return and increased ICP.

Low Vt values can potentially cause hypercapnia and subsequent cerebral vasodilation, leading to an increase in ICP [8,27]. On the other hand, studies suggest that pulmonary protective ventilation is associated with significantly higher survival in patients with TBI complicated by ARDS [8,28]. The use of high Vt values (>9 mL/kg body weight) in patients with TBI may provoke the development of ARDS [27]. Protective ventilation improves the respiratory index in patients with severe TBI (*p* < 0.01) [5]. High PEEP values in ARDS reduce atelectasis and improve PaO_2_ and lung compliance [29]. Most clinicians now tend to use high PEEP values (up to 15 cm wg) in patients with TBI in the absence of intracranial hypertension [30]. The availability of adequate neuromonitoring tools would help establish the ideal PEEP level for such patients. These data are consistent with the results of our study.

Notably, according to Sina Chen (2023) [2], the overall 28-day mortality rate in patients with TBI ranges from 30 to 60%. In our study, the overall 28-day mortality rate is within these limits and is 54.2%. The high values of the indicator can be explained by the fact that only patients with concomitant pathology, severe head injury complicated by ARDS, were included in the study. In our work, we determined the factors that influenced the mortality rate. It was found that mortality does not depend on the level of PaO_2_ in the blood but depends on the neurological status of the patient, assessed by the Glasgow Scale, the respiratory index PaO_2_/FiO_2_, FiO_2_, Vt, PEEP, and the ICP value determined before the start of the analyzed ventilation period (ICP1). The main finding of our study is that the use of a protective ventilation mode significantly (*p* < 0.001), from 78.6% to 31.4%, reduces the mortality of patients with severe TBI complicated by ARDS. In our work, protective ventilation was performed in 51.8% of patients. If the protective strategy had been carried out at an earlier time, even against the background of elevated ICP, it could have provoked a further rise in ICP and an increase in mortality. that in our study, the median ICP before the start of ventilation was 14 mmHg; that is, it did not exceed average values. This is explained by the relatively late timing of determining this indicator, which depended on the onset time of ARDS. On average, this was the middle or end of the second week after surgical treatment. This time was enough for the ICP indicator, which was elevated at the onset of the disease, to normalize. However, since ARDS developed relatively late, when ICP dropped to average values, PEEP in the range from 6 to 15 mm Hg did not increase ICP and mortality. The data indicate that when ventilating patients with severe TBI complicated by ARDS, the presence of lung pathology should be considered, and such patients should be ventilated using a protective strategy. Notably, most deaths were recorded when PEEP was below 5 cm wg or above 15 cm wg. The significant difference in mortality between protective and non-protective strategy can be explained by the more significant therapeutic effect of protective ventilation in ARDS. Mortality from ARDS without TBI is commensurate with the severity of the disease: it is 27%, 32%, and 45% for mild, moderate, and severe ARDS [31]. In our study, the 28-day mortality rate using protective ventilation was 28.5, 28.9, and 45.8%, respectively, in mild, moderate, and severe forms of ARDS. That is, mortality was practically no different from that in isolated ARDS. In most patients, death occurred with average ICP values. At a relatively late date (after the 10th day after surgery), mortality depends primarily on extracranial complications, mainly ARDS. When PEEP increases above 15 cm wg, the increase in mortality can be explained by a likely rise in intracranial pressure and deterioration in neurological status. If we analyze the impact of the PEEP indicator on mortality, notably, patients whose indicator was less than 5 cm wg or more than 15 cm wg died first. According to our data, the optimal PEEP for patients with severe TBI and ARDS is an indicator that ranges from 6 to 15 cm wg. This level of PEEP is high enough to protect the lungs but does not lead to an increase in ICP.

The findings are consistent with a retrospective study by Nemer SN et al., 2015 [32], which showed that PEEP in the range from 0 to 15 cm H_2_O does not affect ICP in patients with severe TBI. In their view, the effect of PEEP on ICP is small if PaCO_2_ is adequately controlled [32], the European Society of Intensive Care Medicine (ESICM) recommendations for MV in patients with TBI for 2023 are significant [23].

The European Consensus recommended avoiding hyperoxia and hypoxia, which are associated with poor prognostic outcomes. Researchers recommended maintaining PaO_2_ at 80–120 mmHg, higher than the usual target range for patients in intensive care units (60–80 mm Hg). The consensus recommendations state that PEEP in patients with severe TBI without ARDS should be set according to the principles considered in the general patient populations in intensive care units. There is only one Consensus recommendation regarding ventilation of patients with severe TBI and concomitant ARDS: in patients without a significant increase in ICP, a protective mechanical ventilation strategy should be used (strong recommendation, with no evidence). Our data fully supports this recommendation.

The findings of this study may help to inform future prospective clinical trials and the refinement of ventilation protocols for patients with concomitant TBI and ARDS. In particular, maintaining moderate PEEP levels and low tidal volume while carefully monitoring intracranial dynamics may represent a practical approach to improve outcomes in this high-risk population.

## 5. Study Limitations and Future Directions

This study has several limitations that should be acknowledged. Its retrospective multicenter design limits causal inference and may introduce residual confounding despite standardized data collection via the EMCiMED system. Although missing data were minimal (<5%) and imputed using mean substitution, this approach may slightly reduce data variability and model robustness. The study cohort, while representative of three large neurocritical care units, may not fully capture inter-center differences in ventilation practices or patient characteristics. Another limitation is the absence of external validation of the predictive model, which restricts its generalizability. Moreover, data on cerebral autoregulation and perfusion were not available for all patients, which could affect interpretation of intracranial dynamics. Future research should focus on prospective multicenter trials to confirm these findings and to evaluate the dynamic effects of ventilation strategies on cerebral perfusion and long-term neurological outcomes. Integration of continuous multimodal monitoring (PaCO_2_, brain tissue oxygenation, autoregulation indices) could help refine individualized ventilation targets in severe TBI complicated by ARDS.

## 6. Conclusions

This retrospective multicenter study demonstrates that the use of lung-protective ventilation in patients with severe traumatic brain injury complicated by ARDS is associated with a marked reduction in 28-day mortality—from 78.6% to 31.4%. Daily monitoring of PaCO_2_ and ICP enabled the safe application of higher PEEP levels without increasing intracranial pressure. Hypercapnia, high tidal volume, low GCS score, elevated ICP, and increased lactate and sodium were independently associated with mortality. Septic shock and multiple organ dysfunction, particularly in patients requiring vasopressors or dialysis, further worsened outcomes.

An optimal ventilation strategy for TBI patients with ARDS should integrate both neurological and respiratory priorities. These findings confirm that protective ventilation (tidal volume <8 mL/kg IBW and PEEP > 5 cm H_2_O) can be safely implemented in this population, improving outcomes through optimized gas exchange, controlled ICP, and balanced cerebral perfusion. The results provide a foundation for developing evidence-based ventilation protocols tailored to neurocritical care settings.

Future prospective randomized studies are warranted to validate these findings and to refine individualized ventilation parameters for patients with concomitant TBI and ARDS.

## Figures and Tables

**Table 1:** Standardized treatment protocols applied across the three participating ICUs.

Treatment Aspect	Unified Protocol (All Centers)	Possible Inter-Center Variations
Mechanical ventilation	Volume-controlled mode, Vt < 8 mL/kg IBW, PEEP 6–10 cm H_2_O, FiO_2_ adjusted to SpO_2_ > 92%	Initial FiO_2_ 0.4–0.5 depending on physician judgment
ICP management	Head elevation 30°, normothermia, osmotherapy (mannitol 20%, hypertonic saline 3%), continuous ICP monitoring	Frequency of monitoring updates
Sedation and analgesia	Propofol 1–4 mg/kg/h ± fentanyl 1–2 µg/kg/h; daily sedation interruption when feasible	Choice between midazolam or dexmedetomidine for long-term sedation
Hemodynamic support	Maintain MAP > 70 mmHg; norepinephrine as first-line vasopressor; balanced crystalloids for fluid therapy	Dose titration and weaning strategy
Antibiotic prophylaxis	Empiric broad-spectrum antibiotics (ceftriaxone ± metronidazole) adjusted after cultures	Duration 5–7 days depending on local policy
ARDS management	Lung-protective strategy, recruitment maneuvers if PaO_2_/FiO_2_ < 150 mmHg, prone positioning if tolerated	Frequency of proning sessions
Neurological assessment	GCS and pupillary reactivity recorded at least 3 times daily	Additional neuroimaging as indicated
Nutrition	Enteral feeding within 48 h after admission	Type of formula (standard vs. immune-modulating)
DVT and ulcer prophylaxis	LMWH 40 mg SC daily + PPI 40 mg IV daily	Minor timing differences per center

Abbreviations: ICP—intracranial pressure; PEEP—positive end-expiratory pressure; FiO_2_—fraction of inspired oxygen; MAP—mean arterial pressure; GCS—Glasgow Coma Scale.

**Table 2:** Baseline demographic and clinical characteristics of patients with severe TBI and ARDS.

	Minimum	Maximum	Median	QI–QIII
Age, years	18	76	45	40.5–54
BMI, kg/m^2^	26	34	30	29–32
Sex, f	22	74	42	32–56
Sex, m	22	76	44	30–62
PaO_2_/FiO_2_, mmHg	87	288	154	121–187
ICP1, mmHg	14	15	14	14–15
Glasgow1, points	7	8	8	8–8

**Table 3:** Gas exchange parameters, functional state of the central nervous system, and ventilation characteristics during mechanical ventilation (n = 301).

	Minimum	Maximum	Median	QI–QIII
PaO_2_, mmHg	60	142	104	80.75–116.25
FiO_2_, %	0.3	1	0.7	0.5–0.9
Vt, mL/kg	6	9	7	7–8
PEEP, cm H_2_O	3	16	9	4–11
PaCO_2_, mm Hg	34	46	42	35–44
ICP2, mmHg	14	15	14	14–15
Na, mmol/L	132	148	144	134–145
Osmolarity, mOsm/L	274.3	295.28	287.84	276.68–289.7
Ppeak, cm H_2_O	25.0	30.0	30.0	25.0–30.0
Pplateau, cm H_2_O	20.0	25.0	25.0	20.0–25.0
Lactate, mmol/L	0.50	2.20	0.90	0.70–1.40
Glasgow2, points	8	11	8	8–9

**Table 4:** Mortality rate by ARDS severity and ventilation mode.

PaO_2_/FiO_2_	Protective Regime		Non-Protective Strategy		*p*
	N	Mortality	N	Mortality	
PaO_2_/FiO_2_ < 100	24	11 (45.8%)	27	24(88.8%)	<0.001
100 < PaO_2_/FiO_2_ < 200	83	24 (28.9%)	95	76 (80.0%)	<0.001
200 < PaO_2_/FiO_2_ < 300	49	14 (28.5%)	23	14 (60.8%)	<0.001
50 < PaO_2_/FiO_2_ < 300	156	49 (31.4%)	145	114 (78.6%)	<0.001

**Table 5:** Risk analysis of mortality factors in univariate logistic regression models.

Independent Variables		Coefficients of Model, b ± m	The Level of Significance of the Difference of the Coefficient from 0, *p*	OR (95% СІ)
Age, per year		0.020 ± 0.010	0.051	1.02 (1.00–1.04)
Sex	m	Reference		
	f	0.03 ± 0.25	0.895	1.03 (0.64–1.67)
BMI, per kg/m^2^		−0.007 ± 0.068	0.911	0.99 (0.87–1.13)
Glasgow1, points		−4.40 ± 1.02	<0.001	0.01 (0.002–0.09)
PaO_2_/FiO_2_, per 1 mmHg		−0.017 ± 0.003	<0.001	0.98 (0.98–0.99)
PaO_2_, per 1 mmHg		−0.007 ± 0.005	0.173	0.99 (0.98–1.00)
FiO_2_, per 1%		3.16 ± 0.58	<0.001	23.7 (7.58–74.1)
Vt, per 1 mL		2.42 ± 0.30	<0.001	11.3 (6.20–20.5)
PEEP, per 1 cm H_2_O		−0.44 ± 0.05	<0.001	0.65 (0.59–0.71)
ICP1, per 1 mm Hg		1.09 ± 0.29	<0.001	2.98 (1.69–5.27)

**Table 6:** Feature Coefficients (Multivariate Regression).

Feature	Coefficient
PaCO_2_	1.617481
icp2	0.280501
Pplateaur	0.245603
Na	0.172124
Lactat	0.160323
age	0.073761
FiO	0.006876
Vt	0.001013
PaO	−0.01045
PaO/FiO	−0.01375
Ppeak	−0.03233
bmi	−0.12642
Osmolarn	−0.25496
icp1	−0.33134
PEEP	−0.46208
Glasgow1	−0.60095
Glasgow2	−0.73431

## Data Availability

The data presented in this study are available on request from the corresponding author. The data are not publicly available due to privacy and ethical restrictions.

## Supplementary Materials

The following supporting information can be downloaded at: https://www.mdpi.com/article/10.3390/brainsci15111151/s1, Table S1: Between-group comparison of intracranial pressure and neurological status Protective vs. Non-protective ventilation (median [IQR]; Mann–Whitney U-test).
