# Supplementary material for: Efficiency of a Protective Mode of Mechanical Ventilation in Patients with Severe Traumatic Brain Injury Complicated by Acute Respiratory Distress Syndrome
Source: Brain Sci. 2025 Oct 27;15(11):1151. doi: 10.3390/brainsci15111151 (PMC12650321; doi:10.3390/brainsci15111151)
Supplement: Supplementary file 1 [file brainsci-15-01151-s001.zip › brainsci-3915198-supplementary.pdf]

Table S1. Between-group comparison of intracranial pressure and neurological status

Protective vs. Non-protective ventilation (median [IQR]; Mann–Whitney U-test)

Parameter	Protective ventilation median [IQR]	Non-protective ventilation median [IQR]	Between-group p
ICP (mm Hg)	14.00 [14.00–14.00]	14.00 [14.00–15.00]	p = 0.053
GCS (points)	10.00 [9.00–10.00]	8.00 [7.00–8.00]	p = 0.000
ΔGCS (points)	2.00 [1.00–2.00]	0.00 [0.00–0.00]	p = 0.000

Values are presented as median [IQR]. Mann–Whitney U-test was used for between-group comparisons. Group definition: Protective = Vt < 8 mL/kg and PEEP > 5 cm H_2O; Non-protective = otherwise. ICP — intracranial pressure; GCS — Glasgow Coma Scale.

Sample sizes used for comparisons: Protective (n = 127), Non-protective (n = 174).
